# Supplementary material for: One-Day Molecular Detection of Salmonella and Campylobacter in Chicken Meat: A Pilot Study
Source: Foods. 2021 May 19;10(5):1132. doi: 10.3390/foods10051132 (PMC8161052; doi:10.3390/foods10051132)
Supplement: Supplementary file 1 [file foods-10-01132-s001.zip › foods-1227596-supplementary.pdf]

Supporting Material for

One-Day Molecular Detection of *Salmonella* and *Campylobacter* in Chicken Meat: A Pilot Study

Andrea Zendrini [1,2], Valentina Carta [3], Virginia Filipello [3,4,*], Laura Ragni [3], Elena Cosciani-Cunico [3], Sara Arnaboldi [3], Barbara Bertasi [3], Niccolò Franceschi [1], Paolo Ajmone-Marsan [1], Dario De Medici [5] and Marina Nadia Losio [3,4]

[1] Department of Animal Science, Food and Nutrition—DIANA, Università Cattolica del Sacro Cuore. Via E. Parmense, 84, 29122 Piacenza, Italy; a.zendrini@unibs.it (A.Z.); niccolo.franceschi@unicatt.it (N.F.); paolo.ajmone@unicatt.it (P.A.-M.)
[2] Department of Molecular and Translational Medicine, University of Brescia. Viale Europa, 11, 25123 Brescia, Italy
[3] Department of Food Safety, Istituto Zooprofilattico della Lombardia e dell'Emilia Romagna. Via A. Bianchi, 9, 25124 Brescia, Italy; valentina.carta@izsler.it (V.C.); laura.ragni@izsler.it (L.R.); elena.coscianicunico@izsler.it (E.C.-C.); sara.arnaboldi@izsler.it (S.A.); barbara.bertasi@izsler.it (B.B.); marinanadia.losio@izsler.it (M.N.L.)
[4] National Reference Centre for Emerging Risks in Food Safety—CRESA, Istituto Zooprofilattico della Lombardia e dell'Emilia Romagna, Via G. Celoria, 12, 20133 Milan, Italy
[5] Department of Food Safety and Veterinary Public Health, Istituto Superiore di Sanità. Viale Regina Elena, 299, 00161 Rome, Italy; dario.demedici@iss.it
* Correspondence: virginia.filipello@izsler.it; Tel.: +39-030-2290-781

S1. Pathogen plate count

The following tables recap the results of pathogen plate count for *Salmonella* and *Campylobacter*. Results have been divided in 2 tables, one for each batch of minced chicken meat.

Table S1a. Plate Counts for *Salmonella* spp. Samples.

Matrix.	Initial Experimental Contamination (C.F.U./g)	Enrichment Time (h)	Rep	Plate count (Log C.F.U./mL)	
				Batch 1	Batch 2
Control broth	10^5	0	1	4.23	5.23
			2	4.34	5.32
			3	4.15	5.32
		2	1	5.04	5.85
			2	5.15	5.79
			3	5.18	6.00
		4	1	6.68	7.85
			2	6.61	7.81
			3	6.53	7.81
		6	1	8.20	8.81
			2	8.45	8.74
			3	8.40	8.88
Chicken minced meat	10^1	0	1	1.46	1.70
			2	1.46	1.46
			3	1.46	1.46
		2	1	2.38	1.88
			2	2.48	2.15
			3	2.52	1.93

	4	1	3.53	1.46
		2	3.62	2.64
		3	3.59	2.20
	6	1	5.79	4.48
		2	6.00	4.48
		3	6.00	4.48
10^3	0	1	3.26	3.56
		2	3.41	3.48
		3	3.28	3.59
	2	1	4.49	4.00
		2	4.28	4.18
		3	4.73	4.04
	4	1	6.20	6.04
		2	6.51	6.20
		3	6.51	6.20
	6	1	8.08	7.48
		2	8.26	7.63
		3	8.23	7.58
10^5	0	1	5.26	4.94
		2	5.15	4.81
		3	5.15	5.08
	2	1	6.57	5.28
		2	6.58	5.26
		3	6.32	5.45
	4	1	8.11	7.34
		2	8.08	7.18
		3	8.11	7.20
	6	1	9.61	8.77
		2	9.68	8.56
		3	9.64	8.68
Negative control (no contamination)	0	1	<1.00	<1.00
		2	<1.00	<1.00
		3	<1.00	<1.00

Table S1b. Plate counts for *Campylobacter* spp. samples.

Matrix	Initial Experimental Contamination (C.F.U./g)	Enrichment Time (h)	Rep	Pathogen count (Log C.F.U./mL)	
				Batch 1	Batch 2
Control broth	10^5	0	1	4.57	4.67
			2	4.75	4.69
			3	4.53	4.78
		2	1	4.54	4.63
			2	4.18	4.43
			3	4.41	4.54
		4	1	4.18	4.18
			2	4.48	4.23
			3	4.08	4.38
		6	1	4.48	4.34
			2	4.30	4.51

			3	4.54	4.71
Chicken minced meat	10^1	0	1	2.15	2.36
			2	2.11	2.43
			3	2.00	2.51
		2	1	2.68	2.88
			2	2.68	2.86
			3	2.81	2.54
		4	1	2.69	2.41
			2	2.82	2.30
			3	2.54	2.20
		6	1	3.26	2.88
			2	3.15	3.01
			3	3.08	2.51
	10^3	0	1	2.00	2.51
			2	2.15	2.60
			3	1.85	2.51
		2	1	2.91	2.59
			2	3.00	2.81
			3	2.80	2.46
		4	1	3.08	3.00
			2	3.23	2.32
			3	3.15	3.08
		6	1	3.40	3.20
			2	3.48	3.18
			3	3.54	2.91
	10^5	0	1	5.00	4.40
			2	4.43	4.32
			3	4.82	4.60
		2	1	4.67	4.62
			2	4.75	4.53
			3	5.18	4.73
		4	1	5.28	4.64
			2	5.04	4.74
			3	5.20	5.11
		6	1	5.30	4.72
			2	5.04	5.08
			3	5.26	5.18
	Negative control (no contamination)	0	1	<0.30	<0.30
			2	<0.30	<0.30
			3	<0.30	<0.30

S2. Colorimetric LAMP inclusivity test

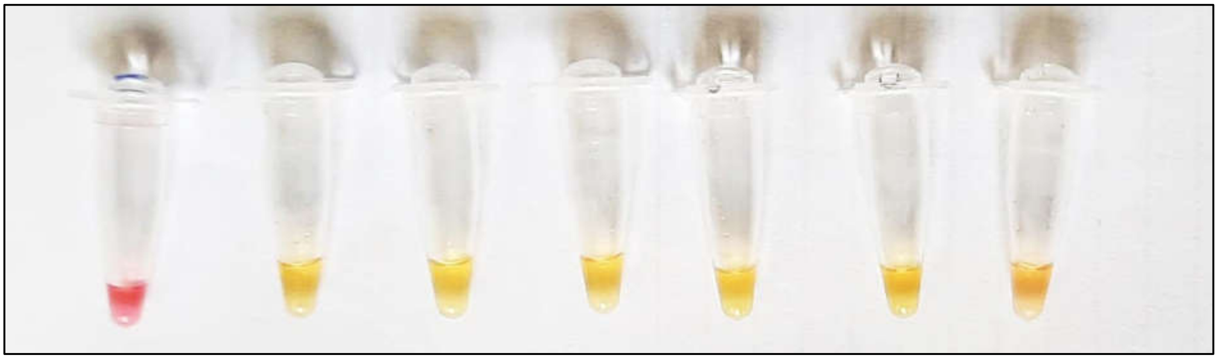

1 2 3 4 5 6 7

Figure S1a. LAMP inclusivity test for Salmonella spp. detection. The sample positivity is verified observing the change of color of the mix (from red to yellow). Legend: 1 = Negative control; 2 = S. Typhimurium ATCC 6994; 3 = S. Enteritidis ATCC 13076; S. Infantis NCTC 6703; field samples: 4 = S. Derby; 5 = S. Newport; 6 = S. Anatum; 7 = S. Typhimurium monophasic variant.

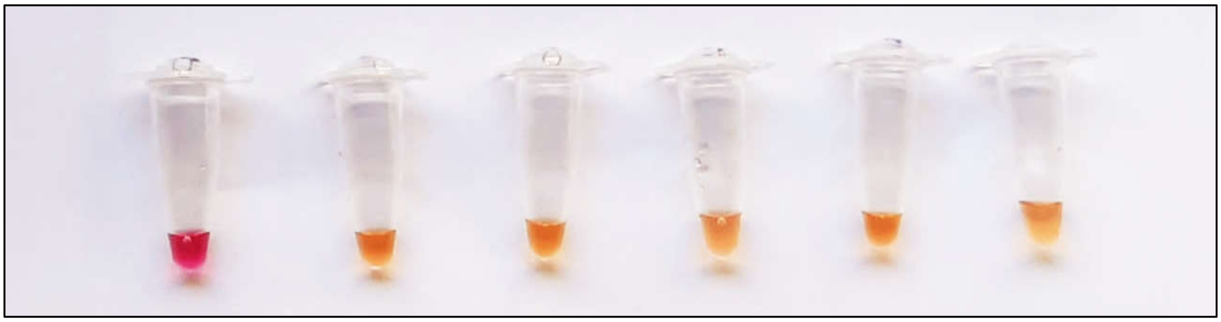

1 2 3 4 5 6

Figure S1b. LAMP inclusivity test for *Campylobacter* spp. detection. The sample positivity is verified observing the change of color of the mix (from red to yellow). Legend: 1 = Negative control; 2 = ATCC 43478; 3 = ATCC 49913; 4 = ATCC 33291; 5–6 = *Campylobacter* spp. field samples.

S3. Summary of RT-PCR and colorimetric LAMP results

The following table summarizes and compares the results of RT-PCR and colorimetric LAMP we performed in the study.

Table S2. Summary of the results regarding RT-PCR and colorimetric LAMP analysis of chicken minced meat samples contaminated with *Salmonella* and *Campylobacter*.

***Salmonella* spp.**			
	colorimetric LAMP		RT-PCR
Time	30 min	45 min	90 min
Samples detected	78/96 (81.3%)	93/96 (96.9%)	89/96 (92.7%)
LOD batch 1	10^1 C.F.U./g T6	10^1 C.F.U./g T0	10^1 C.F.U./g T6
LOD batch 2	10^1 C.F.U./g T4	10^1 C.F.U./g T2	10^1 C.F.U./g T4
***Campylobacter* spp.**			
	colorimetric LAMP		RT-PCR
Time	30 min	45 min	90 min
Samples detected	71/96 (74%)	82/96 (85.4%)	71/96 (74%)
LOD batch 1	10^3 C.F.U./g T0	10^1 C.F.U./g T4	10^3 C.F.U./g T0
LOD batch 2	10^3 C.F.U./g T0	10^3 C.F.U./g T0	10^3 C.F.U./g T0

S4. RT-PCR datasets

The following tables contains the RT-PCR data used to generate the graphs showed in Figure 3 and 5 respectively.

Table S3a. Dataset used to plot the graphs showed in Figure 3.

Salmonella					
		Batch 1		***Batch 2***	
Sample	T	ΔCt	Avg	ΔCt	Avg
Broth (C^+)	0	30.12	30.19	24.34	25.00
		30.1		25.54	
		30.01		25.12	
	2	26.44	26.59	24.81	24.86
		26.68		24.78	
		26.65		24.98	
	4	24.25	24.48	19.49	18.60
		24.24		18.72	
		24.94		17.58	
	6	23.57	23.31	16.85	17.40
		22.65		17.82	
		23.71		17.54	
10^1 C.F.U./g	0	NA	37.47	NA	NA
		37.83		NA	
		37.11		NA	
	2	38.13	38.01	NA	NA
		38.33		NA	
		37.57		NA	
	4	33.5	33.14	36.67	36.31
		33.23		34.7	
		32.69		37.58	
	6	29.38	29.23	31.33	32.68
		29.18		33.34	
		29.13		33.38	

10^3 C.F.U./g	0	33.97	33.60	32.37	34.08
		33.9		35.22	
		33.15		34.65	
	2	32.06	31.94	32.7	32.55
		32.37		30.14	
		31.4		34.81	
	4	25.64	26.70	26.32	27.36
		26.83		27.04	
		27.62		28.73	
	6	23.46	23.70	22.11	22.61
		24.14		22.67	
		23.51		23.06	
10^5 C.F.U./g	0	31.07	30.99	30.98	31.04
		31.22		31.12	
		30.68		31.03	
	2	25.39	25.20	30.04	28.74
		25.62		27.32	
		24.58		28.85	
	4	25.3	25.26	23.04	23.37
		25.15		23.86	
		25.28		23.2	
	6	22.86	22.67	20.6	19.62
		22.28		19.01	
		22.88		19.24	

Table S3b. Dataset used to plot the graphs showed in Figure 5.

		Campylobacter			
		Batch 1		***Batch 2***	
Sample	T	ΔCt	Avg	ΔCt	Avg
Broth (C^+)	0	25.66	25.54	31.51	31.34
		25.48		31.5	
		25.48		31.01	
	2	25.33	25.48	31.21	31.08
		25.45		31.21	
		25.67		30.82	
	4	25.27	25.33	31.36	31.29
		NA		31.28	
		25.39		31.23	
	6	25.4	25.41	31.17	31.18
		25.41		31.31	
		25.41		31.07	
10^1 C.F.U./g	0	27.33	NA	NA	NA
		NA		NA	
		NA		NA	
	2	NA	NA	NA	NA
		NA		NA	
		NA		NA	
	4	NA	NA	NA	NA
		NA		NA	
		NA		NA	

	6	NA	NA	NA	NA
		NA		NA	
		NA		NA	
10^3 C.F.U./g	0	34.72	35.66	36.91	37.59
		35.92		37.71	
		36.34		38.16	
	2	35.28	35.57	37.89	37.63
		36.02		38.9	
		35.41		36.09	
	4	35.73	35.08	38.65	38.23
		34.48		37.82	
		35.03		N/A	
	6	34.24	36.08	37.71	38.13
		38.25		37.17	
		35.75		39.5	
10^5 C.F.U./g	0	29.2	29.48	32.54	32.41
		29.74		32.48	
		29.51		32.22	
	2	26.47	27.90	32.55	32.64
		28.57		32.61	
		28.65		32.75	
	4	27.1	27.40	32.46	32.38
		27.56		32.46	
		27.55		32.21	
	6	31	28.43	32.51	32.31
		28.06		32.2	
		26.23		32.21	

S5. Colorimetric LAMP panels

In this section, the whole data regarding colorimetric LAMP are reported. Each batch of contaminated chicken minced meat was tested for the presence of *Salmonella* or *Campylobacter* DNA using short (30 min) and long (45 min) colorimetric LAMP reaction. Each positive sample was measured in triplicate, with a total of 96 measurements per batch.

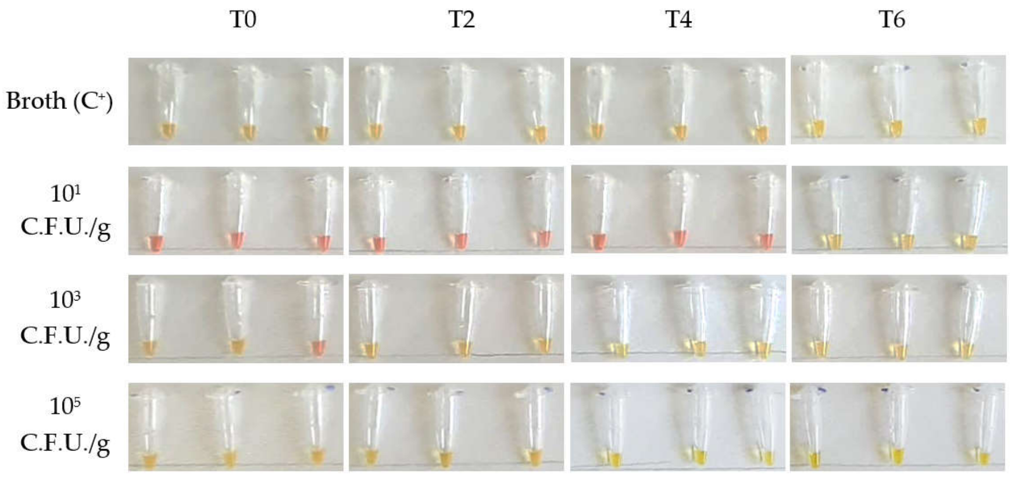

***Salmonella*, batch 1, 30-minutes**

Figure S2. Detection of *Salmonella* DNA in the first batch of contaminated chicken meat using 30-minutes-long colorimetric LAMP. The panel shows all the 96 samples of the batch. Negative samples are red, positive samples are yellow.

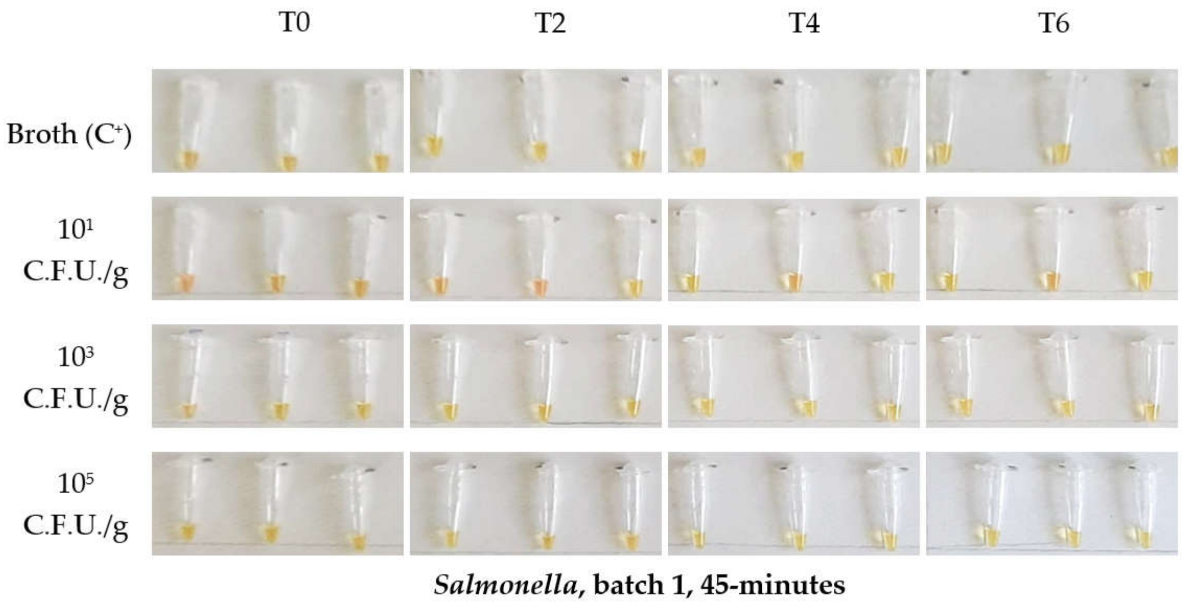

Figure S3. Detection of *Salmonella* DNA in the first batch of contaminated chicken meat using 45-minutes-long colorimetric LAMP. The panel shows all the 96 samples of the batch. Negative samples are red, positive samples are yellow.

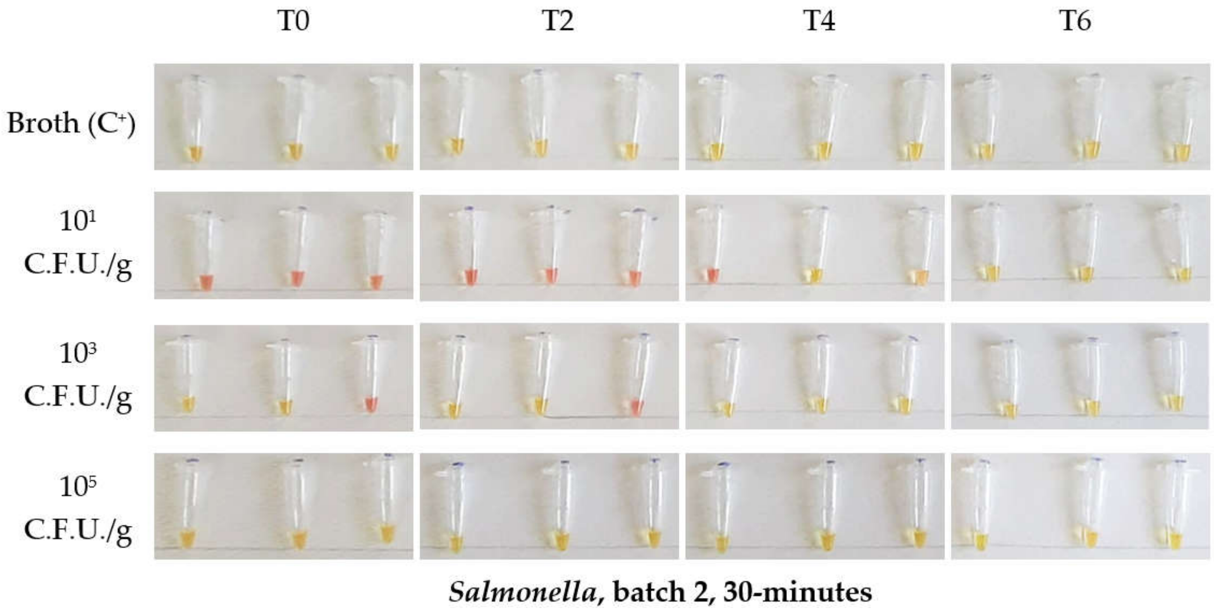

Figure S4. Detection of *Salmonella* DNA in the second batch of contaminated chicken meat using 30-minutes-long colorimetric LAMP. The panel shows all the 96 samples of the batch. Negative samples are red, positive samples are yellow.

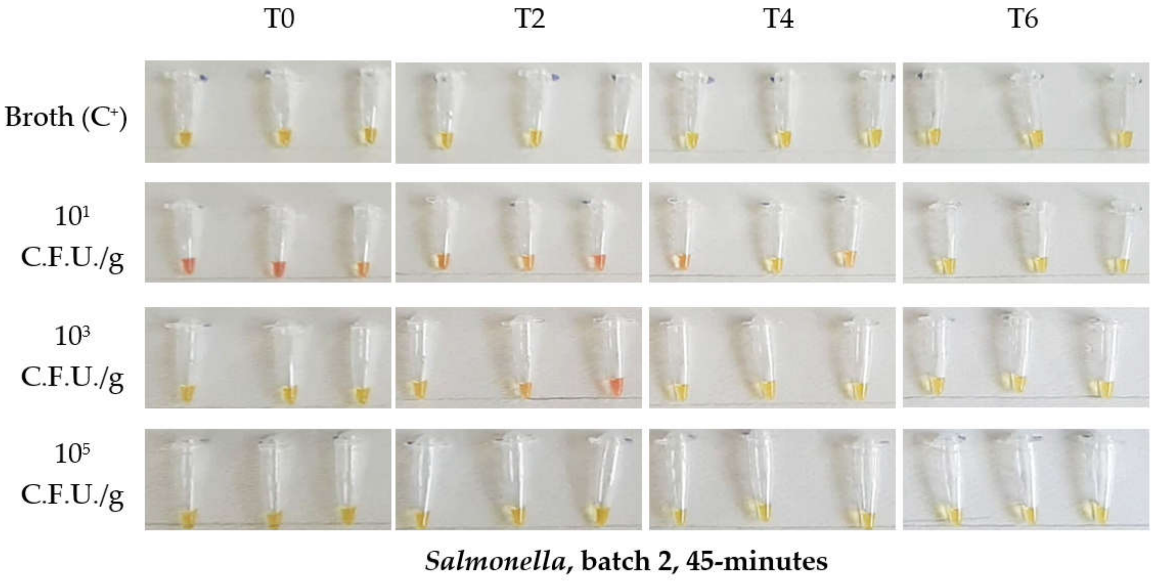

Figure S5. Detection of *Salmonella* DNA in the second batch of contaminated chicken meat using 45-minutes-long colorimetric LAMP. The panel shows all the 96 samples of the batch. Negative samples are red, positive samples are yellow.

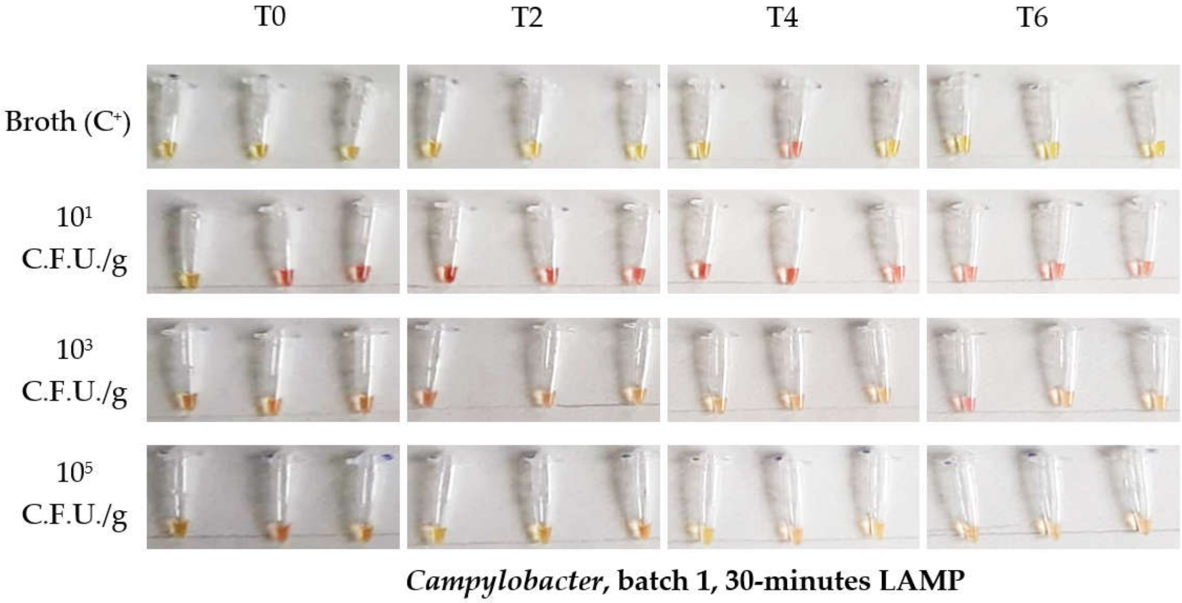

Figure S6. Detection of *Campylobacter* spp. DNA in the first batch of contaminated chicken meat using 30-minutes-long colorimetric LAMP. The panel shows all the 96 samples of the batch. Negative samples are red, positive samples are yellow.

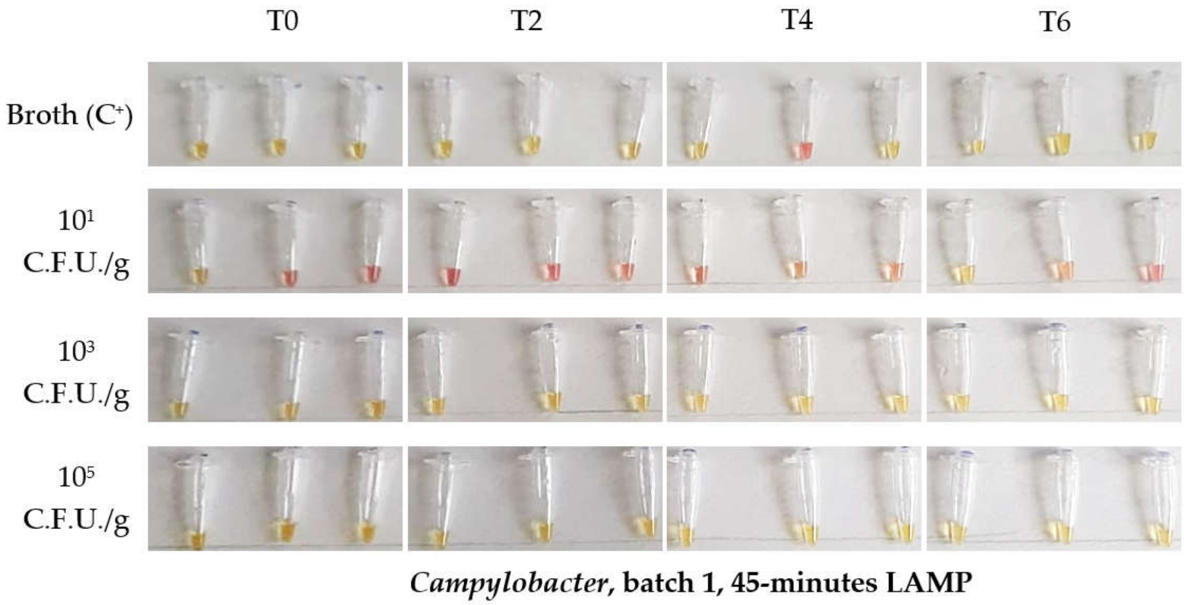

Figure S7. Detection of *Campylobacter* spp. DNA in the second batch of contaminated chicken meat using 45- minutes-long colorimetric LAMP. The panel shows all the 96 samples of the batch. Negative samples are red, positive samples are yellow.

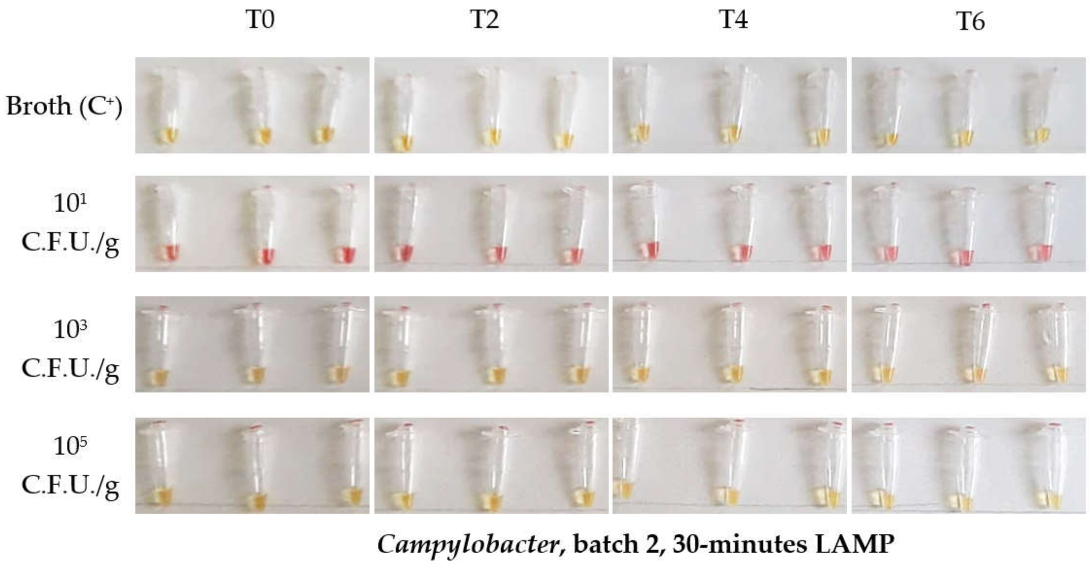

Figure S8. Detection of *Campylobacter* spp. DNA in the second batch of contaminated chicken meat using 30- minutes-long colorimetric LAMP. The panel shows all the 96 samples of the batch. Negative samples are red, positive samples are yellow.

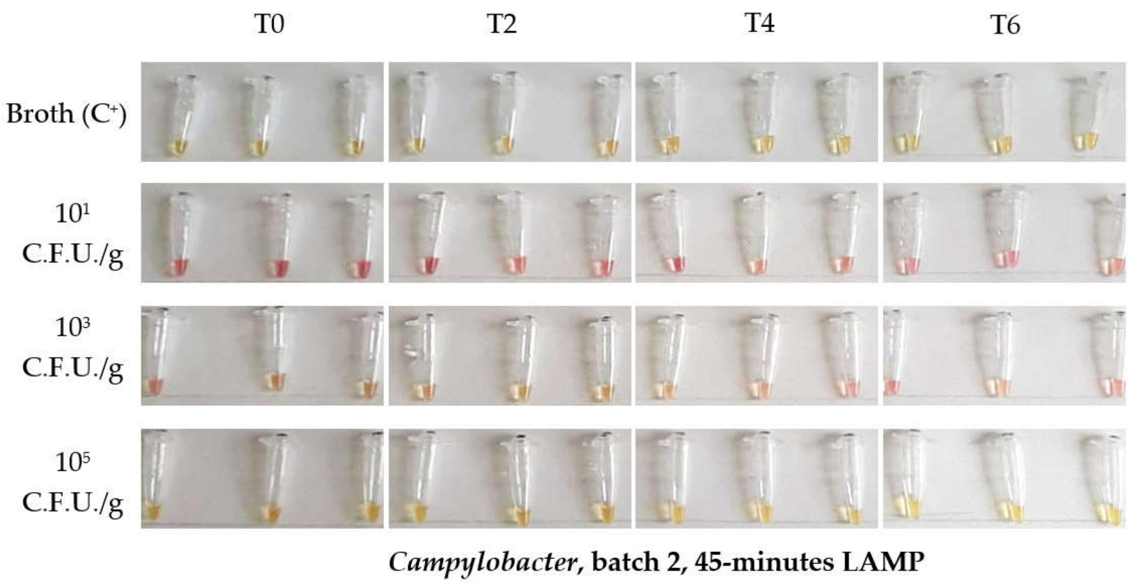

Figure S9. Detection of *Campylobacter* spp. DNA in the second batch of contaminated chicken meat using 40- minutes-long colorimetric LAMP. The panel shows all the 96 samples of the batch. Negative samples are red, positive samples are yellow.
